# Diversity and distribution of 16S rRNA and phenol monooxygenase genes in the rhizosphere and endophytic bacteria isolated from PAH-contaminated sites

**DOI:** 10.1038/srep12173

**Published:** 2015-07-17

**Authors:** Anping Peng, Juan Liu, Wanting Ling, Zeyou Chen, Yanzheng Gao

**Affiliations:** 1 Institute of Organic Contaminant Control and Soil Remediation, College of Resource and Environmental Sciences, Nanjing Agricultural University, Nanjing 210095, P.R. China

## Abstract

This is the first investigation of the diversity and distribution of 16S rRNA and phenol monooxygenase (PHE) genes in endophytic and rhizosphere bacteria of plants at sites contaminated with different levels of PAHs. Ten PAHs at concentrations from 34.22 to 55.29 and 45.79 to 97.81 mg·kg^−1^ were measured in rhizosphere soils of *Alopecurus aequalis* Sobol and *Oxalis corniculata* L., respectively. The diversity of 16S rRNA and PHE genes in rhizosphere soils or plants changed with varying PAH pollution levels, as shown based on PCR-DGGE data. Generally, higher Shannon-Weiner indexes were found in mild or moderate contaminated areas. A total of 82 different bacterial 16S rRNA gene sequences belonging to five phyla; namely, Acfinobacteria, Proteobacteria, Chloroflexi, Cyanophyta, and Bacteroidetes, were obtained from rhizosphere soils. For the 57 identified PHE gene sequences, 18 were excised from rhizosphere bacteria and 39 from endophytic bacteria. The copy numbers of 16S rRNA and PHE genes in rhizosphere and endophytic bacteria varied from 3.83 × 10^3^ to 2.28 × 10^6^ and 4.17 × 10^2^ to 1.99 × 10^5^, respectively. The copy numbers of PHE genes in rhizosphere bacteria were significantly higher than in endophytic bacteria. Results increase our understanding of the diversity of rhizosphere and endophytic bacteria from plants grown in PAH-contaminated sites.

Polycyclic aromatic hydrocarbons (PAHs) are a class of typical organic pollutants comprised of two or more benzene rings^1^. PAHs are of great concern due to their mutagenic and carcinogenic potential. Once released into the environment, PAHs are sorbed to the organic fraction of soils^2^. PAHs can enter the food chain through plant uptake of these hydrophobic contaminates from soil, which can damage human health and endanger the ecological environment. Utilizing PAH-degrading bacteria to remedy PAH-contaminated soils and reduce plant PAH pollution risk has attracted much attention in recent years^3^,^4^,^5^. Rhizospheric and endophytic bacteria have PAH biodegradation potential and are widely distributed in the organs and rhizosphere of plants growing in PAH-polluted sites^6^,^7^,^8^,^9^.

Endophytic bacteria reside within the interior tissues of plants without causing harm to either the host plant or the environment^10^. Several endophytic bacteria and functional genes play important roles in degrading PAH-pollutants^8^,^11^,^12^. Investigating the ecological structure and diversity of these endophytic bacteria and PAH-degrading genes in contaminated sites is particularly important for their application in biological remediation. Our previous study revealed that PAH pollution can significantly impact the distribution and community structure of endophytic bacteria in plants from PAH-contaminated sites^13^, but we did not explore PAH-degrading genes at such sites.

Rhizosphere bacteria are isolated from rhizosphere soil^14^, and can increase host plant tolerance to abiotic stress by improving their nutritional status^15^, inhibiting plant disease^16^, and degrading toxic xenobiotic substances^6^. Previous studies have shown that plant species^17^, metabolic types, and environmental conditions^18^ can have specific effects on rhizosphere bacterial communities. The community structures of rhizosphere bacteria in heavy metal- or pesticide-contaminated environments have been investigated^19^,^20^,^21^. Pritchina *et al.*^22^ used a terminal restriction fragment length polymorphism (TRFLP) technique to investigate the rhizosphere bacterial communities of four types of plant grown in the presence of different PAH-contaminated soils; the results showed that the level of PAH pollution had a more significant influence on the rhizosphere bacterial community structure than did the type of host plant. Rhizosphere width (distance from root surface) also affected the bacterial species and quantities^23^. However, limited information is available on the relationship between rhizosphere bacteria and endophytic bacteria in PAH-polluted sites.

Microorganisms can adapt to organic pollution stress by regulating the expression of biodegradation-related genes^24^. Monooxygenase plays an important role in microbial biodegradation of PAHs^25^. The phenol monooxygenase (PHE) gene and several other aromatic oxygenase genes (e.g., toluene/naphthalene/chlorobenzene dioxygenase and ring hydroxylating monooxygenases) are typically used as indicator genes because of their substrate specificity, high conservation, and being the rate-limiting component in aromatic hydrocarbon biodegradation^22^,^26^,^27^,^28^. Therefore, investigations of PAH-degrading genes as well as the diversity and distribution of rhizospheric and endophytic bacteria in PAH-contaminated sites is particularly important; this is also a prerequisite for use of PAH-degradable bacteria to eliminate soil PAH contamination and reduce plant PAH risk.

In this report, we selected two plants; namely, *Alopecurus aequalis* Sobol *(A. aequalis) and Oxalis corniculata* L. *(O. corniculata)*, because they predominated at a PAH-contaminated site. Using a denaturing gradient gel electrophoresis (PCR-DGGE) method combined with fluorogenic quantitative PCR (FQ-PCR) technology, the diversity and distribution of 16S rRNA and phenol monooxygenase genes of rhizospheric and endophytic bacteria in these two plants were studied for the first time. The results increase our understanding of bacterial ecological functions and contributions to the defense against PAH pollution stress and reduction of PAH pollution risk.

## Results

In this study, 10 PAHs designated by the US Environmental Protection Agency as priority pollutants were detected in rhizosphere soils (Table 1): naphthalene (NAP), acenaphthylene (ANY), fluorine (FLU), phenanthrene (PHA), fluoranthene (FLA), pyrene (PYR), benz[a]anthracene (BaA), chrysene (CHR), benzo[b]fluoranthene (BbF), and benzo[ghi]perylene (BghiP). The PAH concentrations in rhizosphere soils of both plants decreased with increasing distance from the aromatics factory. Among them, NAP and PHA were the primary PAHs in the rhizosphere soils of both *A. aequalis* and *O. corniculata*, accounting for 76.51 to 76.97% and 65.35 to 86.87% of the total concentrations, respectively. Interestingly, regardless of the PAH pollution level, the rhizosphere soils of *O. corniculata* accumulated greater quantities of PAHs; the contents of PAHs were 1.34-, 1.35-, and 1.77-fold, respectively, higher than *A. aequalis* rhizosphere soil from the Q to A area.

Excluding BbF and BghiP, the remaining nine PAHs found in rhizosphere soils were also detected in the roots and shoots of both plants (Table 2). As the concentration of PAHs increased in rhizosphere soil (from Q to A), the accumulation of PAHs in plants also increased, the overwhelming majority of which accumulated in roots (Supplementary Figure S1). Similar to rhizosphere soils, the PAH-accumulating ability of *O. corniculata* was greater than that of *A. aequalis*; 1.66 to 2.36- and 1.42 to 1.64-fold higher in roots and shoots at the various sampling sites. Among detectable PAHs, the concentrations of NAP and PHA in both plants were higher, e.g., the total concentrations of NAP plus PHA accounted for 75.03 to 77.97% and 39.40 to 47.74% of the total PAH concentrations in roots and shoots of *A. aequalis*, respectively.

### Detection of 16S rRNA genes of rhizosphere bacteria using PCR-DGGE

**Figure 1:**
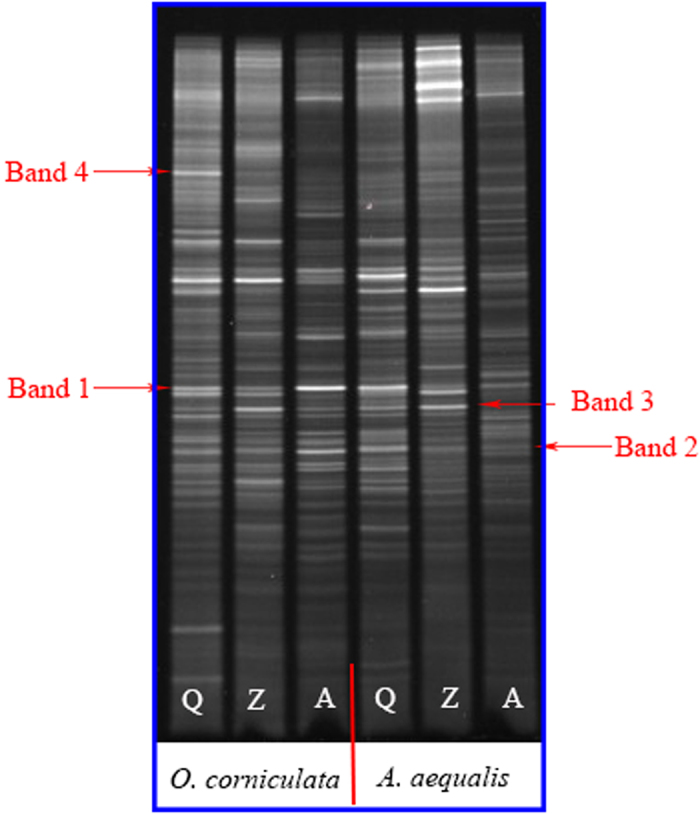
DGGE gel of the 16S rRNA gene of rhizosphere bacteria.

PCR-DGGE profiles of 16S rRNA genes in rhizosphere bacteria are shown in Fig. 1. The presence of ≥30 detectable bands in each sample suggested that a significant number of species of rhizosphere bacteria were present in PAH-contaminated rhizosphere soils. Bands 1 and 2 were detected in all samples, indicating that the rhizosphere bacteria represented by these two bands predominated in rhizosphere soils of both plants. However, the species, distribution, and diversity of bacteria in rhizosphere soils differed according to the rhizosphere soil and PAH pollution level. For example, Band 3 was dominant in rhizosphere soils of *A. aequalis* at low and moderate PAH-contaminated sites (Q and Z), but not at high PAH-contaminated sites (A). Band 4 only presented in rhizosphere soils of *O. corniculata* at site Q. Overall, compared to sites A and Z (corresponding to higher levels of PAH contamination), the rhizosphere soil samples collected from a lightly contaminated area (Q site) had a greater number of bands, which indicated that the increasing PAH contamination level decreases the number of rhizosphere bacterial species. This is supported by the more prominent dominant bands at areas with moderate or high level PAH pollution (Z and A sites).

**Figure 2:**
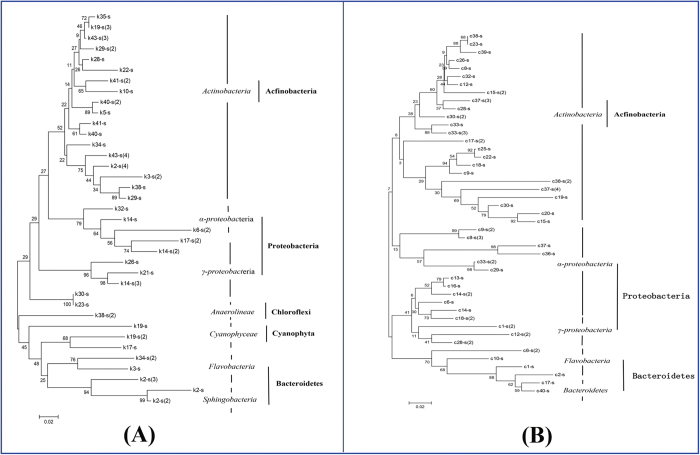
Phylogenetic trees based on 16S rRNA gene analysis of rhizosphere bacterial community of (**A**) *A. aequalis* and (**B**) *O. corniculata*.

The preponderance of dominant bands in the DGGE gel were sequenced, and 82 different bacterial sequences were obtained after removing the 16S rRNA and 18S rRNA genes of mitochondria and chloroplasts, among which 37 were isolated from *A. aequalis* rhizosphere soils and 45 from *O. corniculata* rhizosphere soils. Phylogenetic analysis showed that the total bacterial sequences were classified into five phyla (Acfinobacteria, Proteobacteria, Chloroflexi, Cyanophyta, and Bacteroidetes) and eight classes (Actinobacteria, α-proteobacteria, γ-proteobacteria, Anaerolineae, Cyanophyceae, Flavobacteria, Sphingobacteria, and Bacteroidetes) (Fig. 2). The majority of isolates from the rhizosphere soils of the two plant species belonged to Actinobacteria (48.6% of the sequences isolated from *A. aequalis* and 53.3% of those isolated from *O. corniculata*), followed by Proteobacteria (21.6% and 33.3% for the two rhizosphere soils).

### Detection of the phenol monooxygenase gene in rhizospheric and endophytic bacteria using PCR-DGGE

**Figure 3:**
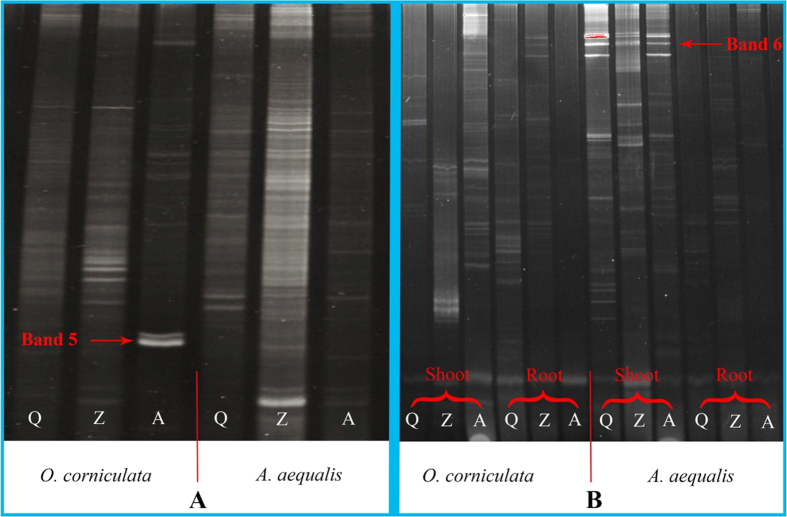
A DGGE gel of the PHE gene from (**A**) rhizosphere bacteria and (**B**) endophytic bacteria.

**Figure 4:**
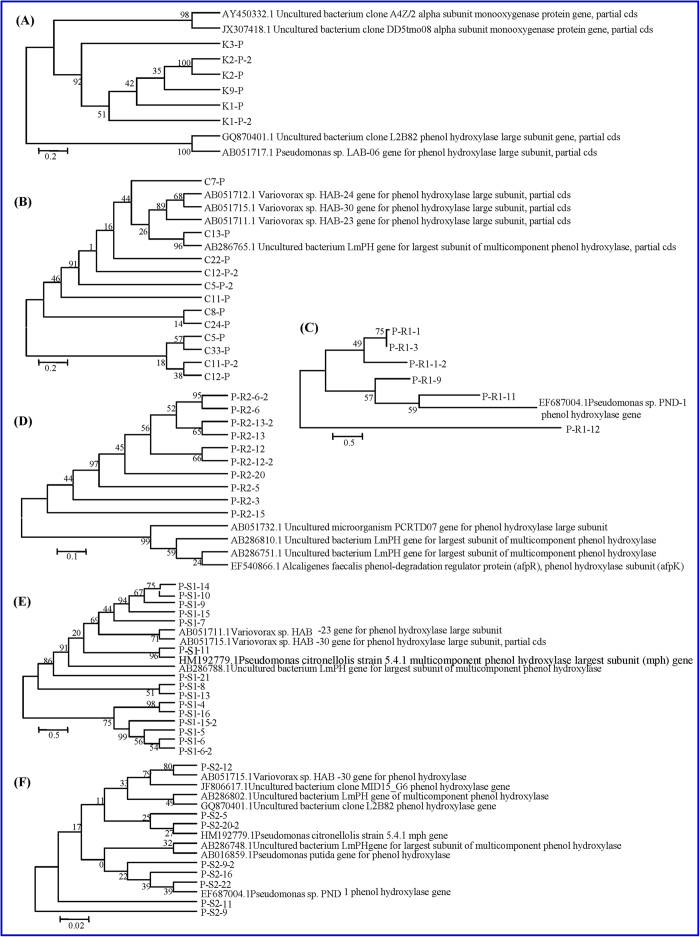
Phylogenetic trees constructed by PHE gene sequence analysis of the rhizosphere bacterial community of (**A**) *A. aequalis* and (**B**) *O. corniculata* and endophytic bacterial community: (**C**) Root of *A. aequalis*; (**D**) Shoot of *A. aequalis*; (**E**) Root of *O. corniculata*; (**F**) Shoot of *O. corniculata*.

The phenol monooxygenase (PHE) gene was detected in rhizosphere and endophytic bacteria at the various PAH-polluted sites (Fig. 3). Using the DGGE fingerprint atlas, we can see that all detected samples contained dominant bands. After excising these bands from the gel, purification, cloning, sequencing, and using the NCBI database and nucleotide BLAST (http://blast.ncbi.nlm.nih.gov/Blast.cgi), we showed that these bands represented the phenol monooxygenase gene. The data in Fig. 3 suggest that plant type, plant tissue, and PAH-pollution levels significantly impact the distribution of PHE genes in both rhizosphere and endophytic bacteria; this is similar to the 16S rRNA gene in rhizosphere bacteria. For example, band 5 was present only in rhizosphere bacteria of *O. corniculata* at site A, and band 6 was observed only in endophytic bacteria in the shoot of *A. aequalis*. In total, 57 bands were sequenced and analyzed, among which 18 were excised from rhizosphere bacteria (*A. aequalis* - 6; *O. corniculata* - 12), and 39 from endophytic bacteria (*A. aequalis* - 21; *O. corniculata* - 18) (Fig. 4). These 57 sequenced bands closely matched phenol monooxygenase gene sequences in the GenBank database with high sequence identities (86 to 95%). The bands detected were derived from *Pseudomonas* sp., *Variovorax* sp., *Alcaligenes* sp., and uncultured bacteria.

To explore the diversity of 16S rRNA and PHE genes in rhizosphere and endophytic bacteria, Shannon-Weiner and Simpson indexes were calculated (Table 3). The Shannon-Weiner indexes of 16S rRNA and PHE genes in rhizosphere bacteria were similar among the various pollution levels, with values ranging from 3.25 to 3.45 and 3.06 to 3.39, respectively. However, greater variation in the diversity of endophytic bacteria according to pollution level was detected; i.e., the Shannon-Weiner index of 16S rRNA and PHE genes in endophytic bacteria ranged from 2.55 to 3.40 and 1.34 to 2.42, respectively. Compared with *A. aequalis*, the diversity of endophytic bacteria in *O. corniculata* was higher at almost all sampling positions (0.87 to 1.12-fold higher), but the diversity of PHE genes was lower (0.80 to 0.94-fold higher). A lower Shannon-Weiner index but a higher Simpson index for endophytic than rhizosphere bacteria at the same pollution level in most situations could be due to the presence of considerably greater numbers of rhizosphere bacteria under certain circumstances^29^. Furthermore, the high concentrations of PAHs in rhizosphere soils may contribute to this difference.

### The 16S rRNA gene copies in rhizospheric and endophytic bacteria

**Figure 5:**
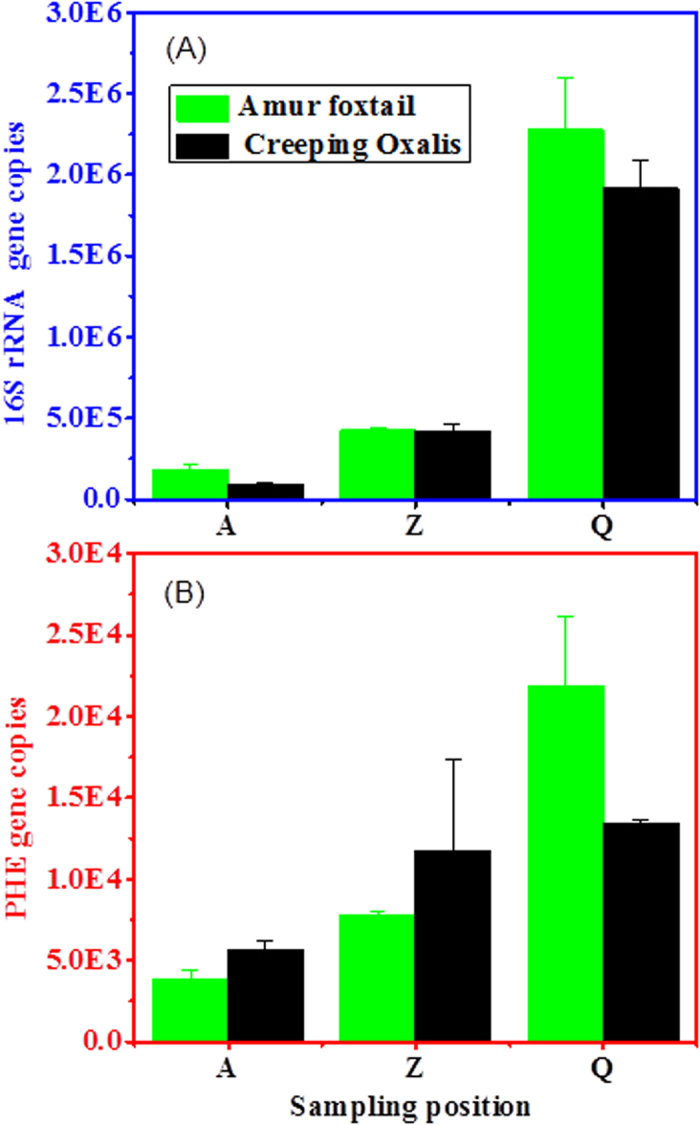
(**A**) 16S rRNA and (**B**) PHE gene copy numbers in rhizosphere bacteria of two plant species as determined by FQ-PCR.

In recent years, use of FQ-PCR to analyze the formation and quantitative distribution of bacteria and genes has attracted extensive attention^30^,^31^. The 16S rRNA and PHE gene copy numbers in rhizosphere and endophytic bacteria determined using FQ-PCR are shown in Fig. 5 and Table 4. There was a marked difference between the 16S rRNA and PHE gene copy numbers in rhizosphere and endophytic bacteria. For rhizosphere bacteria, the 16S rRNA gene copy numbers were considerably higher than the PHE gene copy numbers in both plants (16.31 to 142.34-fold higher depending on the pollution levels). In addition, the copy numbers of both genes decreased with increasing PAH pollution. For example, at position A, the 16S rRNA gene copy numbers in *A. aequalis* and *O. corniculata* were 5.69 × 10^5^ and 2.26 × 10^7^, respectively. At position C, the 16S rRNA gene copy numbers increased to 4.84 × 10^6^ and 1.04 × 10^8^, respectively, for the two plants. Comparing the two types of plants, the 16S rRNA gene copy numbers in rhizosphere bacteria of *A. aequalis* were higher than those in *O. corniculata* at all sampling positions, while for PHE genes at the Q and Z sites, the rhizosphere bacteria of *O. corniculata* carried more PHE genes than *A. aequalis*, but at the A site, the number of PHE gene copies was up to 2.31-fold higher for *A. aequalis* than *O. corniculata*.

Similar to rhizosphere bacteria, the copy numbers of the two genes in endophytic bacteria differed significantly according to plant tissue and PAH concentration (Table 4). The copy numbers of 16S rRNA gene in shoot tissues were less than that in root tissues, regardless of the PAH pollution level; however, the opposite trend was observed for the PHE gene. For the same plant tissues, the 16S rRNA gene copy numbers in *A. aequalis* grown at medium-pollution sites were highest (2.93 to 26.00- and 19.28 to 81.56-fold higher than at the other two sites in roots and shoots, respectively). However, the highest endophytic bacteria 16S rRNA gene copy numbers in *O. corniculata* were observed at light pollution areas (Q site), which were 4.10 to 4.62- and 1.47 to 3.21-fold higher than plants at A and Z sites in roots and shoots, respectively. For both plants, the PAH-degrading gene (PHE gene) copy numbers in endophytic bacteria increased with decreasing PAH content, and at the same sampling site the shoots commonly carried a greater number of PHE gene copies than roots.

## Discussion

In this study, both *A. aequalis* and *O. corniculata* showed PAH uptake and accumulation ability, similar to previous reports^13^. At different PAH pollution levels, the RCF (defined as the ratio of the PAH concentration in roots to that in soils on a dry weight basis^32^) and SCF (defined as the ratio of the PAH concentration in shoots to that in soil on a dry weight basis) of total PAHs for *A. aequalis* were 0.399 to 0.450 and 0.097 to 0.108, respectively, while the RCF and SCF of total PAHs for *O. corniculata* were 0.490 to 0.603 and 0.100 to 0.123, respectively. The root tissues accumulated more PAHs from soils than shoot tissues at all sampling positions (Table 3), indicating that the PAHs in plant tissues are derived predominantly from root uptake from PAH-contaminated soils. This result is consistent with those reported by Gao and Zhu^33^, who investigated the capacity of 12 plant species to absorb and accumulate two PAHs (PHE and PYR). They found that SCFs of all plants accounted for 12.0% to 17.9% and 1.74% to 2.70% of RCFs for PHE and PYR, respectively. Compared with *A. aequalis*, *O. corniculata* has greater PAH accumulation ability, which was consistent with previous reports^13^. The RCFs and SCFs of *O. corniculata* at different positions were 122.93% to 134.12% and 92.51% to 120.56% higher than those in *A. aequalis*, respectively.

Previous studies have shown that endophytic bacteria are mainly derived from soil bacteria because the latter can pass into the plants through root fractures^34^, natural openings or wounds^35^, and seeds^36^, and then spread to the stems, leaves, and other organizations on the ground^37^. In this study, the majority of detected rhizosphere bacteria belonged to Actinobacteria and Proteobacteria, which is similar to the results of our study on the community structure of endophytic bacteria in the same two plant species^19^. To some extent, this result supported the concept that rhizosphere bacteria are the prime source of endophytic bacteria. Seghers *et al.*^38^ found that the community structure of endophytic bacteria in *Zea mays* L. was identical to that of the rhizosphere bacteria, and the number of bacteria in root was considerably greater than that in shoot. In our report, the 16S rRNA gene copy numbers in endophytic bacteria in plant roots were considerably higher than that in plant shoots (3.62 to 23.8- and 20.6 to 57.3-fold higher in *A. aequalis* and *O. corniculata*, respectively).

Using the DGGE method, the distribution and diversity of bacteria were measured using the Shannon-Weiner and Simpson indices. Tang *et al.*^39^ investigated the influence of heavy metals and PCBs on the microbial community of paddy soils and showed that the level of heavy metal and PCB pollution had a minor influence on bacterial community structure in paddy soils. Hassanshahian^40^ also found that bacteria isolated from uncontaminated sites have higher diversity compared to those from contaminated sites. In the present work, the diversities of rhizosphere bacteria were not significantly affected by PAH pollution levels. This finding is similar to the results of Altimira *et al.*^41^, who found that although the DGGE profiles of the bacteria changed under long-term Cu-pollution conditions, the Shannon-Weiner index was not affected. However, the diversities of endophytic bacteria and the PHE gene in rhizospheric and endophytic bacteria were affected by PAH pollution levels, which was consistent with previous reports^19^,^42^. Hendrickx^42^ *et al.* suggested that diversity of the BETX-degrading gene was determined by environmental conditions, including the contamination level.

Monooxygenases are a class of enzymes that play an important role in catalytic hydroxylation of aromatic compounds^43^, and the consequence of this biodegradation process is the production of phenolic intermediate products. In this study, the distributions and diversity of the phenol monooxygenase (PHE) gene in rhizosphere and endophytic bacteria of *A. aequalis* and *O. corniculata* grown in PAH-contaminated sites were investigated. A certain level of environmental contaminants can promote the expression of catabolism-related genes responsible for the biodegradation of these pollutants. Altimira *et al.*^41^ showed that copper-resistance genes were present in the three Cu-polluted soils, but not in the non-polluted soil. Siciliano *et al.*^24^ showed that *alkB*, *ndoB*, *ntdAa*, and *ntnM* genes in plants are abundantly expressed, resulting in production of enzymes related to pollutant degradation, which in turn accelerates the metabolism of organic pollutants in plants. In this study, all plant samples accumulated a certain concentration of PAHs, and the PHE genes were detected in most plant tissues, excluding the shoot of *A. aequalis* and the root of *O. corniculata* in the area of high PAH concentration (A position). With an increase in PAH pollution level, the PHE gene diversity and copy number decreased in most situations; this is inconsistent with the study of Langworthy^44^, who found that the frequencies of PAH-degrading genes (*nahA* and *alkB*) in the presence of moderate to high PAH concentrations increased. This discrepancy may due to the different living environments and the various bacterial species that carried the degrading genes.

One notes that the negative control sites (i.e. sites without PAHs but the plants) will be conducive to better clarify the influence of PAH contamination on the community structure and diversity of endophytic and rhizosphere bacteria in contaminated sites, but these negative control sites were not involved in this report. The main reason is due to the difficulties to find the proper PAH-free control sites not only with the same soil physical and chemical properties but also with the same dominant plants (*A. aequalis* and *O. corniculata*). Unfortunately, these control sites were not found in the sampling field. Despite the lack of negative controls, the changes of the diversity and distribution of 16S rRNA and phenol PHE genes in endophytic and rhizosphere bacteria of test plants contaminated with PAHs indicated that PAH contamination level plays a decisive role in bacterial community structure in PAH-contaminated sites.

The ecological structure and diversity of bacterial communities in anthropogenically polluted environments are good indicators^39^ and important prerequisites for bioremediation of organic contaminants. Screening and isolation of target bacteria and genes from rhizosphere soil or plant tissues, and then using them to remove PAHs from soils^45^, can effectively reduce the risk of plant organic contamination^8^,^46^ and protect food security, as well as human health. In this study, a number of rhizosphere bacteria, endophytic bacteria, and PAH-degrading genes were found in soil and plant samples suffering from long-term PAH pollution, indicating that these microorganisms can tolerate PAH pollution and have considerable potential to degrade PAHs. Further tests are required to explore the application of these isolates in organic pollution remediation technology. The mechanisms involved in gene regulation during the biodegradation of organic pollutants in two types of bacteria require further study. In addition, further studies are required on the interactions between these bacteria and plants. Overall, the insertion of genes associated with degradation of organic pollutants into common bacteria to construct engineered endophytic bacteria, and their induced colonization of host plants might accelerate the metabolism of organic pollutants.

## Conclusions

This is the first investigation into the distribution and diversity of the 16S rRNA gene and PAH metabolism-related genes (phenol monooxygenase) in rhizosphere and endophytic bacteria in two common plants (*A. aequalis* and *O. corniculata*) grown in PAH-polluted sites. The results revealed large numbers of rhizosphere bacteria, endophytic bacteria, and phenol monooxygenase (PHE) genes in rhizosphere soils and host plants. PAH pollution levels can affect the distribution, diversity, and levels of rhizosphere bacteria, endophytic bacteria, and PHE genes in endophytic bacteria. Actinobacteria and Proteobacteria were the two major bacterial types detected, regardless of PAH pollution level. As pollution levels increased, the copy numbers of PHE genes in rhizosphere bacteria decreased significantly, but the diversity was unaffected. These results increase our understanding, and provide an important theoretical basis for application, of plant-related bacteria or PAH-degrading genes to eliminate PAH contamination and reduce the risks associated with plant PAH contamination.

## Methods

### Chemical reagents

A total of 16 PAH standards dissolved in acetonitrile were purchased from Shanghai Anpel Scientific Instruments (Shanghai, China): naphthalene (NAP), acenaphthylene (ANY), acenaphthene (ANE), fluorine (FLU), phenanthrene (PHA), anthracene (ANT), fluoranthene (FLA), pyrene (PYR), benz[a]anthracene (BaA), chrysene (CHR), benzo[b]fluoranthene (BbF), benzo[k]fluoranthene (BkF), benzo[a]pyrene (BaP), indeno[1,2,3-cd] pyrene (InPy), dibenz[a,h]anthracene (DiahA), and benzo[ghi]perylene (BghiP). The concentration of each compound in the acetonitrile mixture was 200 mg•L^−1^. 40% acrylamide / bis-acrylamide solution, 50 × TAE buffer, and ammonium persulfate (APS) were obtained from Nanjing Chemical Reagent Co. Ltd. (Nanjing, China).

### Sample collection

*A. aequalis*, *O. corniculata*, and rhizosphere soils of the two plants were obtained near an aromatics factory in Nanjing. Depending on the distance from the aromatics factory (from far to near), the sampling positions were named Q, 20–25 m; Z, 5–10 m; and A, 0.3–0.5 m, respectively. The physicochemical characteristics of the rhizosphere soils were as follows: pH, 5.87; 13.0% sand, 60.7% silt, 26.3% clay, and 1.36% organic matter. Plant samples were removed from the soil, carefully placed into a plastic bag, and immediately transported to the laboratory. Rhizosphere soil collection was performed as described by Barillot *et al.*^47^, with minor modifications. Briefly, after shaking the plants by hand 5 min to remove bulk soil, rhizosphere soil was collected by shaking the roots for 10 min. Next, plants were gently rinsed with fresh water to remove adherent soil.

### PAH analysis

Some of the rhizosphere soils and plant samples were freeze-dried immediately to determine the PAHs contents. PAHs were exacted from soil and plant samples, as described previously by Ling and Gao^26^. The concentrations of PAHs were analyzed using high-performance liquid chromatography (HPLC) with a reverse-phase C18 column (Inertsil ODS-SP, 5 μm, 4.6 × 150 mm, GL Sciences Inc., Japan) using gradient elution.

### Surface sterilization

The plant samples were surface sterilized as described previously by Sobral^48^ with minor modifications. Roots and shoots tissues were rinsed five times with deionized water, immersed in 75% (v/v) ethanol for 3–5 min, washed with sterile water three times, immersed in 2% (v/v) sodium hypochlorite for 3 min, and rinsed with 75% ethanol for 30 s. Finally, the samples were washed three times with sterilized distilled water to remove surface sterilization agents. To determine whether the sterilization process was successful, the sterile distilled water used in the final rinse was plated onto fresh beef extract peptone agar plates and incubated at 28 °C for 7 days to detect any remaining epiphytic bacteria.

### PCR-DGGE analysis

Total DNA extraction of rhizosphere bacterial community was performed using the Fast DNA Spin Kit for Soil (MP Biomedicals, USA) and total DNA extraction of endophytic was performed according to the protocol described by Garbeva *et al.*^49^. The 16S rDNA V3 sequences were amplified by PCR using extracted genomic DNA as a template; the PCR mixture (25.0 μL) contained 1 μL of DNA template (5 ng·μL^−1^), 12.5 μL of Premix Taq (TaKaRa, Premix Taq Version 2.0), 0.5 μL of primers (12.5 μg·μL^−1^), and 1 μL of bovine serum albumin (20 μg·μL^−1^). PCR was performed in a DNA Engine Thermal Cycler (TaKaRa, D-8308), and the PCR primers and programs were provided in the Supplementary Table S1-S2 online.

DGGE analysis was performed using a Dcode Multiple System (Bio-Rad Laboratories Inc., Hercules, CA, USA) using the following protocol: Aliquots (25 μL) of the PCR products were loaded onto an 8% (w/v) polyacrylamide gel with a denaturant gradient ranging from 40% to 65% (16S rRNA gene) and from 45% to 75% (PHE gene). Electrophoresis was performed for 12 h at 120 V and 60 °C in 0.5 × TAE buffer (20 mM Tris, 10 mM acetic acid, and 1 mM EDTA). Next, gels were soaked in SYBR Green I nucleic acid stain (1:10,000 dilution) for 30 min and immediately photographed under UV light. Specific bands were excised from the DGGE gel and washed twice with sterilized distilled water. Each band was used as a direct template for PCR to recover DNA fragments separated in a previous round of DGGE. The PCR conditions were identical to those used for the original PCR. The fragments recovered from the PCR were subjected to DGGE again to confirm the equality of their mobility. If a single band appeared in a DGGE gel for one sample, the PCR products were purified using the PCR Cleanup Kit (Axygen, USA) and used for direct sequencing (Invitrogen). When multiple bands appeared in one sample, the bands were repeatedly electrophoresed and excised until only a single band was detectable on the DGGE gel.

### FQ-PCR analysis

FQ-PCR analysis was performed using total DNA extracted from rhizosphere bacteria or endophytic bacteria at each sampling site. The experimental system for real-time PCR reaction involved a 20 μL reaction and was performed in a 96-well PCR plate. The reaction primers and reaction conditions were as used for PCR-DGGE analysis. The SYBR Premix Ex Taq™ (Perfect Real Time) kit (TAKARA) was applied in this reaction system. The specific reaction system was as follows: 1 μL of DNA template; 1 μL of each primer (12.5 μg·μL^−1^); SYBR Premix Ex Taq 12.5 μL; Nuclease-free water 9.5 μL. Next, the program was started, run status was monitored, baseline cycles were adjusted and threshold values were calculated, and finally real-time data analysis was performed.

### Statistical analysis

All data collected were processed using Microsoft Excel 2007. Each data point represents the mean of at least three replicates, and error bars represent standard deviations (SD). The data were statistically analyzed using analysis of variance (ANOVA) with the statistical software package SPSS 13.0. Differences were considered significant at *p* values < 0.05, and standard deviations obtained from three parallel samples are shown in the figures as error bars.

## Additional Information

**How to cite this article**: Peng, A. *et al.* Diversity and distribution of 16S rRNA and phenol monooxygenase genes in the rhizosphere and endophytic bacteria isolated from PAH-contaminated sites. *Sci. Rep.* **5**, 12173; doi: 10.1038/srep12173 (2015).

## Supplementary Material

Supplementary Information

## Figures and Tables

**Table 1:** Concentrations of PAHs in: rhizosphere soils (mg·kg^−1^ dry weight).

PAHs	**The rhizosphere soils of** ***A. aequalis*** **(mg**•**kg**^**−1**^ **dry weight)**			**The rhizosphere soils of** ***O. corniculata*** **(mg•kg**^**−1**^**dry weight)**		
	**A**	**Z**	**Q**	**A**	**Z**	**Q**
NAP	32.12 ± 3.18a	26.99 ± 4.04b	21.77 ± 5.43c	51.24 ± 2.89a	32.65 ± 3.96b	29.68 ± 2.37c
FLU	1.89 ± 0.04a	0.99 ± 0.02b	0.79 ± 0.21c	3.12 ± 0.06a	2.8 ± 0.64b	2.15 ± 0.32c
ANY	0.88 ± 0.02b	0.80 ± 0.47b	0.96 ± 0.01a	5.65 ± 0.37a	3.66 ± 0.04b	3.05 ± 0.23b
PHA	10.34 ± 1.89a	7.34 ± 0.59b	4.57 ± 0.75c	12.68 ± 1.71a	9.96 ± 1.66b	4.35 ± 0.23c
FLA	2.85 ± 0.43a	2.13 ± 0.21b	1.35 ± 0.04c	4.63 ± 0.41a	1.25 ± 0.09b	0.79 ± 0.02c
CHR	4.17 ± 0.60a	3.67 ± 0.26b	1.99 ± 0.03c	10.39 ± 0.65a	4.06 ± 0.41b	1.31 ± 0.01c
PYR	0.86 ± 0.04b	0.94 ± 0.01a	0.92 ± 0.02ab	3.33 ± 0.19a	1.89 ± 0.02b	1.53 ± 0.02b
BaA	0.67 ± 0.03a	0.53 ± 0.00b	0.51 ± 0.00b	2.46 ± 0.31a	1.63 ± 0.08b	1.62 ± 0.07b
BbF	0.77 ± 0.00a	0.8 ± 0.05a	0.67 ± 0.00b	3.74 ± 0.04a	1.99 ± 0.05b	0.8 ± 0.00c
BghiP	0.74 ± 0.13a	0.68 ± 0.11b	0.69 ± 0.01b	0.57 ± 0.02a	0.67 ± 0.00a	0.51 ± 0.04a
∑PAHs	55.29 ± 5.19a	44.87 ± 6.34b	34.22 ± 7.98c	97.81 ± 10.10a	60.56 ± 9.28b	45.79 ± 5.49c

Note: different letters in the same row for the same plant indicate significant differences (*P* < 0.05).

**Table 2:** Concentrations of PAHs in: plants (mg·kg^−1^ dry weight).

PAHs	**A. aequalis**						**O. corniculata**					
	**Root**			**Shoot**			**Root**			**Shoot**		
	**A**	**Z**	**Q**	**A**	**Z**	**Q**	**A**	**Z**	**Q**	**A**	**Z**	**Q**
NAP	15.76 ± 1.00a	12.65 ± 1.26b	11.51 ± 0.39c	2.50 ± 0.10a	1.41 ± 0.20b	1.13 ± 0.15b	28.90 ± 2.21a	15.68 ± 1.51b	13.92 ± 0.35b	3.16 ± 0.30a	1.42 ± 0.98b	1.34 ± 0.45b
FLU	1.13 ± 0.08a	0.54 ± 0.00b	0.25 ± 0.11c	0.51 ± 0.01a	0.42 ± 0.05b	0.32 ± 0.01c	1.69 ± 0.37a	0.62 ± 0.19b	0.47 ± 0.20c	0.57 ± 0.01a	0.21 ± 0.06b	0.22 ± 0.04b
ANY	1.12 ± 0.03a	1.00 ± 0.01a	0.79 ± 0.04b	0.65 ± 0.07a	0.69 ± 0.12a	0.4 ± 0.04b	1.92 ± 0.40a	1.28 ± 0.15a	1.15 ± 0.02a	0.32 ± 0.01b	0.34 ± 0.07b	0.57 ± 0.17a
ANE	0.83 ± 0.08a	0.65 ± 0.03b	0.5 ± 0.00c	0.76 ± 0.01a	0.47 ± 0.04b	0.38 ± 0.05c	2.09 ± 0.38a	1.41 ± 0.07b	1.21 ± 0.43b	1.33 ± 0.01a	0.96 ± 0.06a	0.32 ± 0.04b
PHA	1.04 ± 0.15a	0.78 ± 0.07b	0.49 ± 0.02c	0.35 ± 0.03a	0.3 ± 0.04a	0.28 ± 0.03b	1.88 ± 0.13a	0.87 ± 0.05c	1.17 ± 0.38b	0.32 ± 0.02a	0.27 ± 0.07b	0.37 ± 0.02a
FLA	0.73 ± 0.04a	0.67 ± 0.05a	0.52 ± 0.01a	0.29 ± 0.03a	0.28 ± 0.02a	0.26 ± 0.02a	1.61 ± 0.40a	0.96 ± 0.15b	0.93 ± 0.02b	0.46 ± 0.02a	0.4 ± 0.01a	0.29 ± 0.00b
CHR	0.82 ± 0.02a	0.7 ± 0.04a	0.53 ± 0.04b	0.45 ± 0.02a	0.4 ± 0.06a	0.39 ± 0.04a	2.31 ± 0.08a	1.08 ± 0.10b	1.41 ± 0.30b	0.59 ± 0.02a	0.35 ± 0.07b	0.37 ± 0.01b
PYR	0.36 ± 0.02a	0.38 ± 0.03a	0.32 ± 0.00a	0.17 ± 0.01a	0.15 ± 0.02a	0.12 ± 0.01a	0.69 ± 0.01a	0.65 ± 0.26a	0.49 ± 0.09b	0.29 ± 0.03a	0.18 ± 0.04b	0.16 ± 0.00b
BaA	0.58 ± 0.03a	0.53 ± 0.02a	0.48 ± 0.04b	0.29 ± 0.02a	0.23 ± 0.01a	0.21 ± 0.01a	1.51 ± 0.34a	1.09 ± 0.05a	0.83 ± 0.03a	0.37 ± 0.03a	0.28 ± 0.03b	0.24 ± 0.00b
∑PAHs	22.37 ± 1.41a	17.9 ± 0.95b	15.39 ± 1.21b	5.97 ± 0.09a	4.34 ± 0.55b	3.49 ± 0.38c	52.69 ± 2.54a	29.7 ± 1.98b	27.62 ± 1.04b	9.77 ± 0.05a	6.15 ± 1.03b	5.63 ± 0.79c

Note: different letters in the same row for the same plant root or shoot indicate significant differences (*P *< 0.05).

**Table 3:** Diversity analysis of 16S rRNA and PHE genes in rhizosphere and endophytic bacteria.

Gene	Diversity index	Plant	**Rhizosphere bacteria**			**Endophytic bacteria in root**			**Endophytic bacteria in shoot**		
			**A**	**Z**	**Q**	**A**	**Z**	**Q**	**A**	**Z**	**Q**
16S rRNA	Shannon-Weiner index	*A. aequalis*	3.25	3.31	3.33	2.93	3.2	3.38	2.74	3.03	2.55
		*O. corniculata*	3.35	3.28	3.45	3.29	3.27	3.34	2.73	2.66	2.78
	Simpson index	*A. aequalis*	0.042	0.04	0.04	0.057	0.043	0.037	0.071	0.055	0.095
		*O. corniculata*	0.037	0.039	0.034	0.039	0.042	0.037	0.082	0.078	0.071
PHE	Shannon-Weiner index	*A. aequalis*	3.13	3.28	3.23	2.01	2.13	1.23	1.68	2.42	2.21
		*O. corniculata*	3.06	3.21	3.39	1.62	1.88	2.05	1.34	2.28	1.96
	Simpson index	*A. aequalis*	0.047	0.042	0.036	0.137	0.054	0.109	0.227	0.102	0.143
		*O. corniculata*	0.054	0.043	0.038	0.08	0.196	0.052	0.08	0.121	0.157

**Table 4:** 16S rRNA and PHE gene copy numbers in endophytic bacteria of two plant species.

Gene		***A. aequalis***		***O. corniculata***	
		**Root**	**Shoot**	**Root**	**Shoot**
16S rRNA	A	5.23 × 10^5^ ± 1.22 × 10^3^	4.61 × 10^4^ ± 5.11 × 10^3^	2.21 × 10^7^ ± 8.93 × 10^5^	5.54 × 10^5^ ± 2.93 × 10^4^
	Z	1.36 × 10^7^ ± 5.52 × 10^4^	3.76 × 10^6^ ± 5.04 × 10^5^	2.49 × 10^7^ ± 5.43 × 10^4^	1.21 × 10^6^ ± 1.91 × 10^4^
	Q	4.64 × 10^6^ ± 4.50 × 10^4^	1.95 × 10^5^ ± 2.72 × 10^3^	1.02 × 10^8^ ± 4.61 × 10^6^	1.78 × 10^6^ ± 2.42 × 10^4^
PHE	A	3.56 × 10^3^ ± 3.22 × 10^2^	N/A	N/A	8.22 × 10^3^ ± 2.97 × 10^2^
	Z	1.47 × 10^2^ ± 9.29	1.96 × 10^3^ ± 7.78	4.84 × 10^3^ ± 1.10 × 10^2^	1.07 × 10^5^ ± 7.39 × 10^2^
	Q	5.64 × 10^3^ ± 6.95	5.73 × 10^3^ ± 1.39 × 10^2^	1.23 × 10^4^ ± 8.91	1.99 × 10^5^ ± 5.07 × 10^3^
