# Supplementary material for: Diversity and distribution of 16S rRNA and phenol monooxygenase genes in the rhizosphere and endophytic bacteria isolated from PAH-contaminated sites
Source: Sci Rep. 2015 Jul 17;5:12173. doi: 10.1038/srep12173 (PMC4505310; doi:10.1038/srep12173)
Supplement: Supplementary Information [file srep12173-s1.pdf]

Supplementary information for:

Diversity and distribution of 16S rRNA and phenol monooxygenase genes in the rhizosphere and endophytic bacteria isolated from PAH-contaminated sites

Anping Peng[a], Juan Liu[a], Wanting Ling, Zeyou Chen, Yanzheng Gao*

Institute of Organic Contaminant Control and Soil Remediation, College of Resource and Environmental Sciences, Nanjing Agricultural University, Nanjing 210095, P.R. China.

[a] A. Peng and J. Liu contribute equality to this paper.

*** To whom correspondence should be addressed:** Yanzheng Gao, Dr/Prof

Institute of Organic Contaminant Control and Soil Remediation

College of Resource and Environmental Sciences

Nanjing Agricultural University

Weigang Road 1, Nanjing 210095

P.R. China

Tel: +86-25-84395019

Fax: +86-25-84395019

Email: gaoyanzheng@njau.edu.cn; gaosoil@163.com

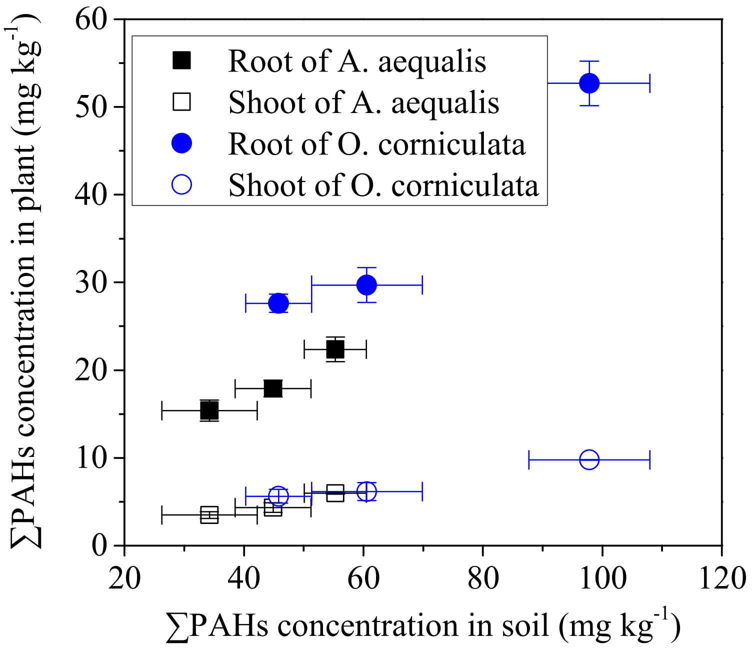

Supplementary Figure S1. Correlations of the total PAH concentrations in soil with those in plants.

Supplementary Table S1. PCR primers

Target	Sequence
16S rRNA V3[1]	with a GC clamp 5'- CGCCCGCCGCGCGCGGCGGGGGGCGGG GGCACGGGGGGCCTACGGGAGGCAGCAG-3'
	5'- ATTACCGCGGCTGCTGG-3'
Phenol monooxygenase[2]	5'-GTGCTGAC(C/G)AA(C/T)CTG(C/T)TGTTC-3'
	5'-CGCCAGAACCA(C/T)TT(A/G)TC-3'

Supplementary Table S2. PCR conditions

Target gene	Initial denaturation		Denaturation		Primer annealing		Extension		Cycles
	temperature (℃)	time (min)	temperature (℃)	time (min)	temperature (℃)	time (min)	temperature (℃)	time (min)	
16S rDNA V3	94	5	94	0.5	60	0.5	72	0.5	30
PHE	95	10	95	1	50	1	72	2	32

References:

1 Yang, G. M., Bao, B. L., Li, H. R., Tang, W. Q. & Ren, D. M. Analysis on the composition of culturable bacterial communities in several tissues of puffer fish *Takifugu obscurus*. *J. Shanghai Ocean Univ.* **17**, 12–21 (2008).

2 Baldwin, B. R., Nakatsu, C. H. & Nies, L. Detection and enumeration of aromatic oxygenase genes by multiplex and real-time PCR. *Appl. Environ. Microbiol.* **69**, 3350–3358 (2003).
